# Screening History, Stage at Diagnosis, and Mortality in Screen-Detected Breast Cancer

**DOI:** 10.1001/jamanetworkopen.2025.5322

**Published:** 2025-04-15

**Authors:** Sida Huang, Sarah J. Westvold, Pamela R. Soulos, Jane Fan, Eric P. Winer, Haiying Zhan, Maryam B. Lustberg, John Lewin, Timothy J. Robinson, Michaela A. Dinan

**Affiliations:** 1 Yale School of Public Health, New Haven, Connecticut; 2 Cancer Outcomes, Public Policy, and Effectiveness Research Center, Yale University, New Haven, Connecticut; 3 Yale Cancer Center, New Haven, Connecticut; 4 Department of Medical Oncology, Yale School of Medicine, New Haven, Connecticut; 5 Department of Pathology, Yale School of Medicine, New Haven, Connecticut; 6 Department of Radiology and Biomedical Imaging, Yale School of Medicine, New Haven, Connecticut; 7 Department of Therapeutic Radiology, Yale School of Medicine, New Haven, Connecticut; 8 Department of Chronic Disease Epidemiology, Yale School of Public Health, New Haven, Connecticut

## Abstract

**Importance:**

Screening mammography promotes early detection of breast cancer and is associated with reduced breast cancer mortality. Screening history prior to diagnosis may impact stage at diagnosis and breast cancer mortality but has not been comprehensively examined within a diverse US cohort.

**Objective:**

To determine whether having a prior screening is associated with earlier stage at breast cancer diagnosis and lower breast cancer–specific mortality.

**Design, Setting, and Participants:**

This cohort study used linked Surveillance, Epidemiology, and End Results–Medicare data of women aged at least 70 years, diagnosed with estrogen receptor–positive or human epidermal growth factor receptor 2–negative breast cancer from 2010 to 2017, and enrolled in fee-for-service Medicare Parts A and B from 5 years prior to through 1 year after diagnosis. Data were analyzed from March 1 to September 18, 2024.

**Exposure:**

Presence of 1 or more screening mammograms during the 5 years prior to the mammogram at breast cancer diagnosis.

**Main Outcomes and Measures:**

Outcomes of interest were stage of breast cancer at diagnosis, dichotomized into very early (T1N0) vs later stage (T2+ or N1+) and breast cancer–specific mortality.

**Results:**

Among 13 028 included women, most had at least 1 prior screening (10 094 women [77.5%]) and were aged between 70 and 79 years (9034 women [69.4%]) and not dual-eligible for Medicare and Medicaid (11 475 women [88.1%]). Additionally, 3812 women (29.3%) were diagnosed with later-stage disease (T2+ or N1+) at the time of diagnosis. In multivariable analyses, prior screening was associated with 54% lower odds of later-stage breast cancer diagnosis (adjusted odds ratio, 0.46; 95% CI, 0.42-0.50) and 36% lower hazard of breast cancer–specific death (adjusted hazard ratio, 0.63; 95% CI, 0.52-0.76) compared with no prior screenin. In the adjusted Cox proportional hazards model, having 3 or 4 prior screenings was associated with 37% reduced hazard of breast cancer–specific mortality compared with having 1 prior screening (adjusted hazard ratio, 0.63; 95% CI, 0.44-0.89).

**Conclusions and Relevance:**

In this cohort study of older women with screen-detected estrogen receptor–positive or human epidermal growth factor receptor 2–negative breast cancer, prior screening mammography was associated with earlier stage at breast cancer diagnosis and lower breast cancer mortality. These findings support the potential for routine screening to improve breast cancer outcomes. As with all observational studies, this study is limited by the potential effects of other differences between the screening and nonscreening groups.

## Key Points

**Question:**

Is screening history prior to breast cancer diagnosis associated with stage at diagnosis and breast cancer–specific mortality among older women?

**Findings:**

In this cohort study of 13 028 women aged 70 years or older with screen-detected breast cancer identified from the Surveillance, Epidemiology, and End Results–Medicare database, women with a history of screening mammography within 5 years prior to breast cancer diagnosis had earlier stage at diagnosis and lower breast cancer–specific mortality compared with women without such screening.

**Meaning:**

In this cohort study among older women with screen-detected breast cancers, prior screening was associated with more favorable stage and mortality; however, the extent to which this reflects a true benefit of screening remains unclear due to potential selection bias.

## Teaser

This cohort study assesses whether having undergone a prior breast cancer screening is associated with earlier stage at breast cancer diagnosis and lower breast cancer–specific mortality.

## Introduction

Routine screening mammography is the most effective means to promote early detection of breast cancer. Biannual screening is recommended for women aged 40 to 74 years by the US Preventive Services Task Force (USPSTF),^1^ as it has been shown to reduce the mortality associated with estrogen receptor–positive (ER+) breast cancer and the incidence of late-stage diagnosis.^2^

However, for the 22 million women in the US aged 75 years and older, there is conflicting evidence as to the benefits of biannual screening.^1,3^ While older women face a reduced survival benefit from early detection due to higher mortality risks from other causes,^4,5^ some benefits of screening extend to them: when breast cancers are detected through a routine mammography, these cancers are often diagnosed at an earlier stage, require less intensive treatment, and have better disease-specific survival.^6,7^ Due to these conflicting findings, the USPSTF notes that there is insufficient evidence to make a recommendation for screening among women aged 75 years and older and cites this as a research need.^1^

One factor that may impact the effectiveness of screening for early detection is screening history, defined by the number and frequency of mammograms received prior to the detection of cancer.^8,9,10,11^ Mammography at routine intervals increases the likelihood of identifying any anomalies or changes in breast tissue as soon as they occur. It may also indicate proactive health behaviors, such as regular engagement with health care services, that further promote the early detection of breast cancer and improved survival. Identifying this potential difference in health outcomes based on screening history may facilitate a more accurate evaluation of screening effectiveness. Previous investigations of the impact of screening history on early detection have been limited in that they included both screen-detected and non–screen-detected cancers, which are known to exhibit differences in stage at diagnosis and prognosis.^12^

This study uses the Surveillance, Epidemiology and End Results (SEER) and Medicare linked dataset to investigate the association between screening history and the breast cancer outcomes among screen-detected cancers within the older US population. We restricted our sample to women with screen-detected breast cancer so that we could isolate the association between screening history and breast cancer outcomes. We hypothesized that women who had received at least 1 screening mammogram within 5 years prior to screening-based detection of breast cancer have a lower risk of being diagnosed with later-stage cancer and have a better survival outcome compared with women with no prior mammogram within the previous 5 years.

## Methods

This cohort study was determined to be exempt from review and informed consent by the Yale School of Medicine institutional review board because SEER-Medicare data were deidentified and informed consent was not required. We followed the Strengthening the Reporting of Observational Studies in Epidemiology (STROBE) reporting guideline.

### Data Source

The study used data from the SEER-Medicare linkage which combines data from the SEER population-based tumor registries with Medicare claims.^13^ The data provide information on the demographics, screening history, cancer diagnosis, biology, and treatment of patients with breast cancer. The data extracted for the analysis covered the period from January 1, 2005, to December 31, 2020. The SEER diagnosis data started on January 1, 2010, while Medicare claims data started on January 1, 2005, to allow for an adequate capture of screening history prior to diagnosis.

### Cohort Selection

**Figure 1.**
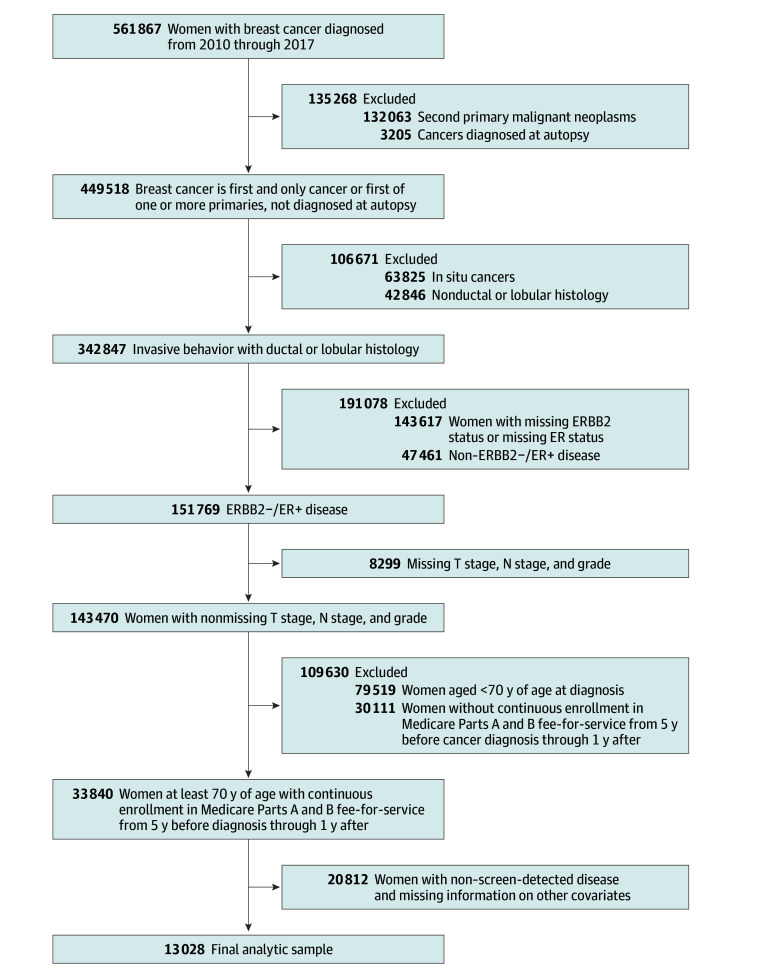
Flowchart for Cohort Construction ER+ indicates estrogen receptor positive; ERBB2−, human epidermal growth factor receptor 2 negative.

This was a retrospective cohort study of women aged 70 years or older diagnosed with ER-positive or human epidermal growth factor receptor 2 (ERBB2)–negative breast cancer between 2010 and 2017 by screening mammogram. We restricted to women aged at least 70 years at diagnosis because Medicare eligibility typically begins at age 65 years and we required 5 years of claims history to evaluate prior screening. These patients were identified using a validated claims-based algorithm that distinguished screening from diagnostic mammograms based on the time to the last mammogram, the presence of diagnostic Healthcare Common Procedure Coding System codes, and whether the patient had claims-based symptoms of breast anomalies within the year prior to the mammogram (eMethods in Supplement 1).^14^ The sample was restricted to women continuously enrolled in Medicare Parts A and B fee-for-service coverage from 5 years prior to diagnosis through 1 year after diagnosis. Continuous enrollment guarantees the complete reporting of health care services that the beneficiary received during this time, which ensures the validity of our claims-based measures. Meanwhile, patients with ductal in situ cancers were excluded due to their distinct prognostic and clinical outcomes compared with invasive breast cancers. Other exclusion criteria are listed in Figure 1.

### Exposure

The exposure was the presence of 1 or more screening mammogram before the mammogram that detected breast cancer during the 5 years before cancer diagnosis. We distinguished screening from diagnostic mammograms during this period using the same aforementioned claims-based algorithm.^14^ For our primary analysis, screening history was dichotomized into no vs any screening prior to diagnosis.

### Outcomes

The primary outcome was stage at diagnosis, dichotomized into very early (T1N0) vs later stage (T2+ or N1+) diagnosis based on American Joint Committee on Cancer TNM stages. This dichotomization was based on the rationale that T2+ or N1+ stages account for approximately 90% of breast cancer–specific mortality despite representing nearly half of all cancers, while T1N0 tumors represent the other half, with substantially lower mortality risk.^15^ The secondary outcome was breast cancer–specific mortality, which was identified by SEER cause of death.

### Covariates

All demographic, socioeconomic, tumor, and treatment-related factors were collected at the time of diagnosis. Demographic factors included age at diagnosis, race and ethnicity, and marital status. Race and ethnicity were obtained from Medicare data, which is based on self-report.^16^ Race and ethnicity were categorized as Asian, Hispanic, non-Hispanic Black, non-Hispanic White, and other or unknown, which included American Indian or Alaska Native and Pacific Islander and those with unreported or missing race and ethnicity. Medicare/Medicaid dual eligibility, which was included as a proxy for socioeconomic status (SES), was defined as at least 1 month of dual eligibility in the year prior to cancer diagnosis. This measure indicates low SES because eligibility for Medicaid is based on having income below certain thresholds, reflecting a certain degree of poverty. Socioeconomic factors measured at the census tract or zip code level included percentage of residents with less than a high school degree and area-level poverty. The year of diagnosis was included to control for the temporal trends in the use of screening, stage at diagnosis, and mortality.

Disease factors included tumor grade and histology. Frailty status was measured in the year before diagnosis using the Kim claims-based frailty index.^17^ Comorbidity in the 2 years before diagnosis was measured using the Elixhauser comorbidity index, and categorized into 0, 1 to 2, and 3 or more comorbidities.^18^

To assess health care utilization and health promoting behaviors, we identified whether patients had any primary care physician (PCP) visits or annual wellness visits (AWV) during the 5 years prior to breast cancer diagnosis (eTable 1 in Supplement 1). Additionally, the number of influenza vaccines received within the same period was categorized into 3 groups: 0, 1 to 2, and 3 or more.^19,20^

### Statistical Analysis

Bivariate and multivariable logistic regression were used to determine the association between socioeconomic factors and primary care use factors and whether the patient had any screening prior to the detection of breast cancer. Logistic regression was then used to evaluate the association between screening history and stage at diagnosis adjusting for other relevant covariates. Cox proportional hazard modeling was used to measure the associations between screening history and breast cancer–specific mortality. Significance was set at *P* < .05, and hypothesis tests were 2-sided.

Covariates significantly associated with outcomes in bivariate analyses were included in the fully adjusted models. Collinearity was assessed using a variance inflation factor greater than 10 with no covariates excluded. The proportional hazard assumption of each variable was tested by plotting the Kaplan-Meier survival function, examining significance of the interaction term of the variable with time and assessing whether the Schoenfeld residuals were significantly correlated with time.

#### Sensitivity Analysis

Previous studies suggest that overdiagnosis may be common among older women diagnosed with breast cancer after screening, complicating the assessment of its benefits.^5^ Therefore, we conducted a sensitivity analysis among women older than 75 years to determine whether the associations between screening history and stage at diagnosis and breast cancer mortality hold in this age group. We conducted a second sensitivity analysis by limiting the cohort to include only patients who received cancer-directed surgery to reflect a curative-intent study population.

#### Additional Analysis

Among women with at least 1 prior screening, we investigated the association between the number of prior screenings and breast cancer outcomes while controlling for the time from the most recent screening. The number of prior screenings was categorized as 1, 2, and 3 or 4 screenings within the 5 years prior to diagnosis. The time to the last screening was defined as the duration between the date of the mammogram that detected the cancer and the date of the most recent mammogram received prior to this, and was dichotomized as being within 2 years vs more than 2 years before the detection.

We also assessed whether prior inconclusive mammograms outside reference ranges were associated with stage at diagnosis and breast cancer specific mortality. Each of the prior screening mammograms was assessed for inconclusive findings that prompted further evaluation using a previously validated claims-based algorithm by Hubbard et al.^21^ The exposure was dichotomized into women with prior screenings without any inconclusive findings and women with prior screenings which included 1 or more inconclusive screening mammograms.

Data were analyzed from March 1 to September 18, 2024. All analyses were conducted using SAS software version 9.4 (SAS Institute) and RStudio software version 2023.06.1 (R Project for Statistical Computing).

## Results

### Cohort Description and Screening History

A total of 13 028 women with screen-detected breast cancer were included in the cohort, including 469 Asian women (3.6%), 552 Hispanic women (4.2%), 803 non-Hispanic Black women (6.2%), 10 986 non-Hispanic White women (84.3%), and 218 women with other or unknown race and ethnicity (1.7%) (Table). Most women were younger than 80 years, with 5015 women (38.5%) aged 70 to 74 years and 4019 women (30.9%) aged 75 to 79 years. Most women were not dual-eligible for Medicare and Medicaid (11 475 women [88.1%]) and had ductal histology (10 228 women [78.5%]). Among the overall study cohort, 10 094 women (77.5%) had prior screening and 2934 women (22.5%) had no prior screening. Among women with prior screenings, 2359 (23.4%) had 1 mammogram, 3132 (31.0%) had 2 mammograms, and 4603 (45.6%) had 3 or 4 mammograms.

**Table.** Baseline Characteristics of the Cohort Subgroup With a History of Screening

Characteristic	Full cohort, No. (column %) (N = 13 028)	With prior screening ≤5 y prior to diagnosis, No. (row %) (n = 10 094)	*P* value^a^
Prior screenings, No.			
0	2934 (22.5)	NA	NA
1	2359 (18.1)	2359 (100)	
2	3132 (24.0)	3132 (100)	
3-4	4603 (35.3)	4603 (100)	
Stage at diagnosis			
Very early stage (T1N0)	9216 (70.7)	7556 (82.0)	<.001
Later stage (T2 or N1+)	3812 (29.3)	2538 (66.6)	
Age at diagnosis, y			
70-74	5015 (38.5)	3966 (79.1)	<.001
75-79	4019 (30.9)	3201 (79.6)	
80-84	2488 (19.1)	1936 (77.8)	
≥85	1506 (11.6)	991 (65.8)	
Race and ethnicity			
Asian	469 (3.6)	356 (75.9)	<.001
Hispanic	552 (4.2)	376 (68.1)	
Non-Hispanic Black	803 (6.2)	577 (71.9)	
Non-Hispanic White	10 986 (84.3)	8614 (78.4)	
Other/unknown^b^	218 (1.7)	171 (78.4)	
Grade			
I	4723 (36.3)	3781 (80.1)	<.001
II	6875 (52.8)	5296 (77.0)	
III and IV	1430 (11.0)	1017 (71.1)	
AJCC stage			
I	9484 (72.8)	7763 (81.9)	<.001
II	2883 (22.1)	1937 (67.2)	
III	534 (4.1)	335 (62.7)	
IV	127 (1.0)	59 (46.5)	
Histology			
Ductal	10 228 (78.5)	7888 (77.1)	.005
Lobular	1919 (14.7)	1539 (80.2)	
Mixed ductal with lobular	881 (6.8)	667 (75.7)	
Medicare/Medicaid dual eligible			
No	11 475 (88.1)	9165 (79.9)	<.001
Yes	1553 (11.9)	929 (59.8)	
Area-level residents in poverty, %			
0 to <5	3217 (24.7)	2607 (81.0)	<.001
5 to 10	3835 (29.4)	3019 (78.7)	
10 to <20	3775 (29.0)	2886 (76.5)	
20 to 100	2201 (16.9)	1582 (71.9)	
Area-level residents with <high school degree, %			
0 to <30	4859 (37.3)	3950 (81.3)	<.001
30 to <40	2472 (19.0)	1949 (78.8)	
40 to <50	2293 (17.6)	1729 (75.4)	
50 to 100	3404 (26.1)	2466 (72.4)	
Marital status			
Single/unmarried	6437 (49.4)	4718 (73.3)	<.001
Married	5991 (46.0)	4934 (82.4)	
Missing	600 (4.6)	442 (73.7)	
Frailty			
Not frail	10 365 (79.6)	8280 (79.9)	<.001
Frail	2663 (20.4)	1814 (68.1)	
Elixhauser comorbidity index			
0	4836 (37.1)	3803 (78.6)	<.001
1-2	5375 (41.3)	4276 (79.6)	
≥3	2817 (21.6)	2015 (71.5)	
Any PCP visit or AWV ≤5 y prior to diagnosis			
No	509 (3.9)	236 (46.4)	<.001
Yes	12 519 (96.1)	9858 (78.7)	
Influenza vaccines ≤5 y prior to diagnosis, No.			
0	2661 (20.4)	1727 (64.9)	<.001
1-2	2101 (16.1)	1507 (71.7)	
≥3	8266 (63.5)	6860 (83.0)	
Receipt of surgery			
Yes	12 628 (96.9)	9912 (78.5)	<.001
No	400 (3.1)	182 (45.5)	
Year of diagnosis			
2010	1607 (12.3)	1263 (78.5)	.80
2011	1635 (12.6)	1275 (77.9)	
2012	1587 (12.2)	1228 (77.3)	
2013	1622 (12.5)	1247 (76.8)	
2014	1555 (11.9)	1184 (76.1)	
2015	1674 (12.9)	1309 (78.1)	
2016	1724 (13.2)	1336 (77.4)	
2017	1624 (12.5)	1260 (77.5)	

Abbreviations: AJCC, American Joint Committee on Cancer; AWV, annual wellness visit; NA, not applicable; PCP, primary care physician.

^a^ *P* values were calculated using Pearson χ^2^ tests to compare the difference of the distribution in covariates between women with prior screening and women without.

^b^ Including American Indian or Alaska Native and Pacific Islander and those with unreported or missing race and ethnicity.

### Factors Associated With Prior Screening

The strongest factors associated with prior screening was access to primary care services. Among women who had at least 1 PCP visit or AWV in the 5 years prior to diagnosis, 9858 (78.7%) had prior screening, compared with only 236 women (46.4%) who did not have such visits (*P* < .001) (Table). In addition, prior screening was more common among younger women (aged 70-74 years; 3966 women [79.1%]) compared with older women (aged ≥85 years: 991 women [65.8%]; *P* < .001) and among non–dual eligible patients (9165 women [79.9%]) compared with dual-eligible patients (929 women [59.8%]; *P* < .001).

**Figure 2.**
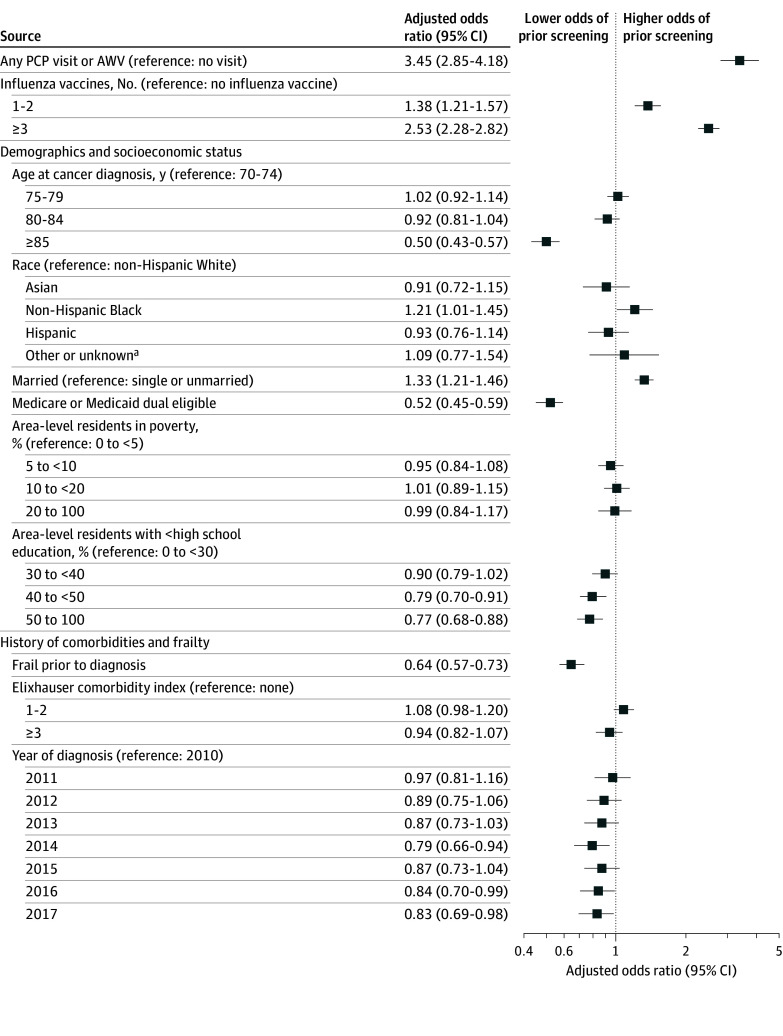
Adjusted Associations of Patient Factors With History of Screening AWV indicates annual wellness visit; PCP, primary care physician. ^a^Including American Indian or Alaska Native and Pacific Islander and those with unreported or missing race and ethnicity.

In the multivariable logistic regression, factors associated with greater odds of prior screening included more primary care use (any PCP visit or AWV vs none: adjusted odds ratio [aOR], 3.45; 95% CI, 2.85-4.18), receiving more influenza vaccines (≥3 vs none: aOR, 2.53; 95% CI, 2.28-2.82), and being married compared with unmarried (aOR, 1.33; 95% CI, 1.21-1.46) (Figure 2). Factors associated with lower odds of prior screening included advanced age (≥85 vs 70-74 years: aOR, 0.50; 95% CI, 0.43-0.57), being dual eligible compared with not dual eligible (aOR, 0.48; 95% CI, 0.43-0.55), lower area-level education (>50% vs 0% to 30% of residents had <high school degree: aOR, 0.77; 95% CI, 0.68-0.88), being frail prior to diagnosis (aOR, 0.64; 95% CI, 0.57-0.73), and being diagnosed in later years compared with 2010 (2017 vs 2010: aOR, 0.83; 95% CI, 0.69-0.98).

### Association Between Prior Screening and Stage at Diagnosis

**Figure 3.**
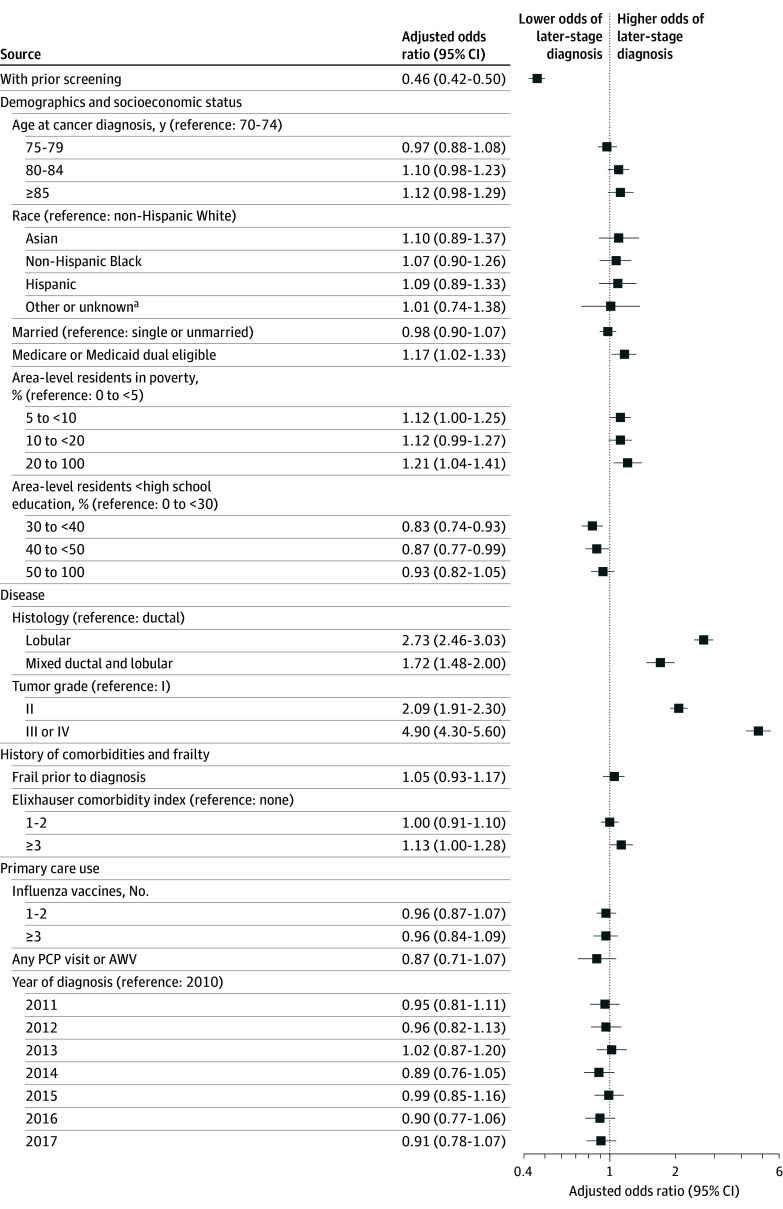
Adjusted Associations of History of Screening and Other Covariates With Stage at Diagnosis AWV indicates annual wellness visit; PCP, primary care physician. ^a^Including American Indian or Alaska Native and Pacific Islander and those with unreported or missing race and ethnicity.

Overall, 3812 (29.3%) women had a later-stage disease (T2+ or N1+) at diagnosis. Among women with prior screening, 2538 (25.1%) were diagnosed at a later stage. In contrast, 1274 women (43.4%) without prior screening were diagnosed at a later stage (*P* < .001) (Table). In the adjusted logistic regression, prior screening was associated with 54% lower odds of being diagnosed with a later-stage disease compared to no prior screening (aOR, 0.46; 95% CI, 0.42-0.50) (Figure 3).

Other factors associated with later-stage diagnosis include being dual eligible compared with not dual eligible (aOR, 1.17; 95% CI, 1.02-1.33). Area-level sociodemographic factors associated with later-stage diagnosis included higher poverty (20%-100% vs 0%-5% of area residents in poverty: aOR, 1.21; 95% CI, 1.04-1.41), and lower education (30%-40% vs 0%-30% of area residents had <high school degree, aOR, 0.83; 95% CI, 0.74-0.93).

Additionally, women diagnosed with lobular histology or mixed invasive ductal-lobular carcinoma (MDLC) had higher odds of being diagnosed at a later stage compared with those with ductal histology (lobular: aOR, 2.73; 95% CI, 2.46-3.03; mixed invasive ductal-lobular carcinoma: aOR, 1.72; 95% CI, 1.48-2.00) (eTable 2 in Supplement 1). Tumors with higher grades were also associated with higher odds of later-stage diagnosis (grade III or IV vs grade I: aOR, 4.90; 95% CI, 4.30-5.60).

### Association Between Prior Screening and Breast Cancer Mortality

**Figure 4.**
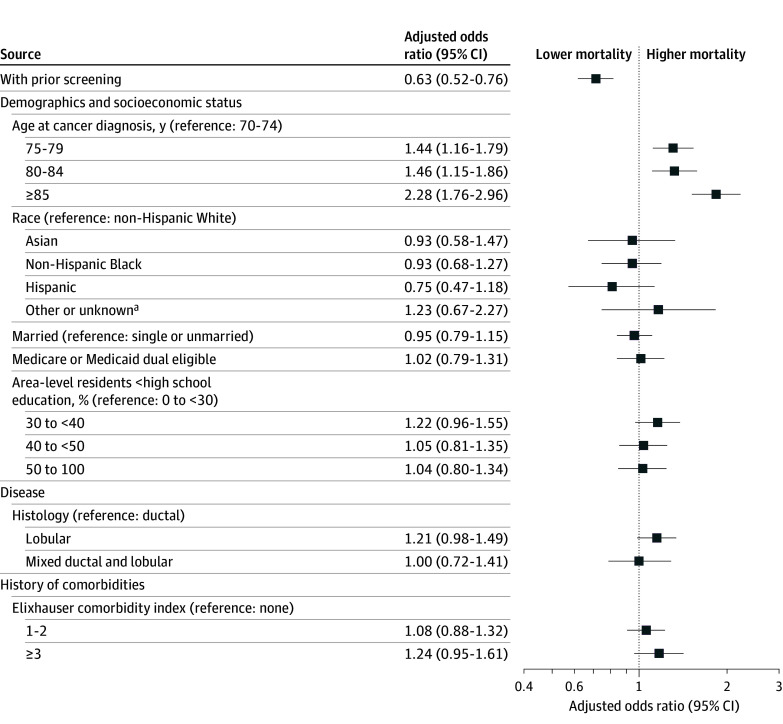
Adjusted Associations of History of Screening With Breast Cancer–Specific Mortality The model was adjusted for American Joint Committee on Cancer overall stage, tumor grade, receipt of surgery, frailty status, any primary care physician (PCP) visit or annual wellness visit (AWV), number of influenza vaccines, area-level poverty, and year of diagnosis, in addition to the covariates in the figure. Hazard ratios of these covariates were not reported due to the violation of the proportional hazard assumption. However, stage, tumor grade, receipt of surgery, frailty prior to diagnosis, and area-level poverty had a statistically significant association with breast cancer–specific mortality. ^a^Including American Indian or Alaska Native and Pacific Islander and those with unreported or missing race and ethnicity.

The main exposure, prior screening, met the proportional hazard assumption for breast cancer–specific survival. Prior screening was significantly associated with breast cancer mortality: a total of 569 deaths from breast cancer (4.4% of the full cohort) were observed, including 234 deaths (8.0%) among women with no prior screening and 335 deaths (3.3%) among women with prior screening (*P* < .001). Prior screening was independently associated with a reduced hazard of breast cancer–specific death compared with no prior screening (aHR, 0.63; 95% CI, 0.52-0.76) (Figure 4).

Older women had a higher hazard of breast cancer–specific death compared with those aged 70 to 74 years (≥85 years: aHR, 2.28; 95% CI, 1.76-2.96). Other covariates that were significantly associated with breast cancer mortality are not reported due to the violation of proportional hazard assumption.

### Sensitivity Analysis

In the sensitivity analyses limited to women older than 75 years, prior screening remained significantly associated with lower odds of being diagnosed with later-stage disease (aOR, 0.41; 95% CI, 0.36-0.46) and lower hazard for breast cancer–specific death (aHR, 0.56; 95% CI, 0.45-0.69) (eTable 3 in Supplement 1). Additional sensitivity analysis limited to women who underwent surgery resulted in similar associations (eTable 4 in Supplement 1).

### Additional Analysis

Among women with prior screenings, having 2 or 3 to 4 prior screenings were each associated with lower odds of later-stage diagnosis compared with having 1 prior screening (2 screenings: aOR, 0.73; 95% CI, 0.63-0.84; 3-4 screenings: aOR, 0.68; 95% CI, 0.58-0.79) (eTable 5 in Supplement 1). In the adjusted Cox proportional hazard model, having 3 to 4 prior screenings was associated with lower breast cancer–specific mortality compared with having 1 prior screening (aHR: 0.63; 95% CI, 0.44-0.89). The time from most recent screening was not significantly associated with stage at diagnosis or breast cancer–specific mortality in the adjusted models. No association was found between having an a prior inconclusive screening result and stage at diagnosis or breast cancer–specific mortality compared to those without prior inconclusive screening results (eTable 6 in Supplement 1).

## Discussion

In this cohort study, we found that routine breast cancer screening, as defined by having a history of 1 or more negative screenings within 5 years prior to breast cancer diagnosis, was associated with almost half the odds of being diagnosed with later-stage diseases and two-thirds the hazard of breast cancer–specific mortality. These associations were consistent among women older than 75 years and those who underwent surgery, with similar effect sizes. Among women with a history of prior screening, more prior screenings were associated with better breast cancer outcomes after adjusting for the time from the most recent screening. These results support the potential benefit of continued routine screening among older women.

We also observed that screening history was correlated with social determinants of health and health care access, including Medicare/Medicaid dual eligibility, area-level education, and primary care use. Patients with lower SES and less engagement with health care services were less likely to have received prior screenings. Although racial and ethnic disparities in screening history were not evident in our study due to limited statistical power, previous research has confirmed that individuals from minoritized racial and ethnic groups (eg, Black, non-White Hispanic) are less likely to receive prior screening mammograms.^22,23^

Our findings are consistent with previous evidence that routine screening mammography is associated with detection of breast cancer at an earlier stage and lower breast cancer mortality, even among older women.^8,9,10,11^ One study of women aged 67 years and older reported that patients with no mammogram during the 2 years prior to diagnosis had 3 times the odds of a later-stage diagnosis and more than 3 times the hazard of experiencing breast cancer mortality compared with those with screening during the same period.^8^ However, previous studies did not consider the impact of detection mode (screen-detected vs symptomatic) on breast cancer outcomes. Our approach of focusing on women with screen-detected breast cancer minimizes confounding from non–screen-detected cancers, which are often more aggressive compared with the screen-detected ones.^12^ Consequently, our results likely offer a more accurate estimate of the impact of screening history.

Our sensitivity analysis suggests an association between screening history and improved outcomes for women older than 75 years, with effect sizes similar to those observed in women aged 70 to 74 years. These findings support the potential benefit that women older than 75 years may experience from continued screening. However, these findings may be partially influenced by selection bias, as women with a prior screening tend to be younger, have better SES, and have more primary care use—factors known to be associated with improved health outcomes. The observed benefits of prior screening were challenged by our additional analysis of the association between number of prior screenings, time to the most recent screening, and breast cancer outcomes. After adjusting for the time to the most recent screening, women with more prior screenings still had improved outcomes, indicating that they may have better overall health. Notably, however, the lack of a significant association between time from most recent screening and outcomes may be due to limited variation in screening intervals. We found that more than 80% of patients with prior screening had their most recent screening in the 2 years prior to the mammogram that detected their cancer, leaving an insufficient number of patients with prolonged screening gaps.

In addition, the benefits of regular screening in older women have been challenged by studies investigating the potential harms of overdiagnosis. One study estimated that 31% of the screen-detected breast cancer among women aged 70 to 74 years may never be clinically significant in their lifetime, with this figure rising to 54% among those aged 85 years or older.^5^ Overdiagnosis can lead to unnecessary tests and overtreatment, causing risks such as emotional distress, scarring, and infections.^24^

Our findings highlight the importance of considering screening history when evaluating the impact of regular mammography among older women. Accounting for a 5-year screening history in older women could better capture the nuanced relationship between screening programs and breast cancer outcomes, ensuring that their associations are evaluated independently of the healthier baseline characteristics of women with a prior screening.

### Limitations

Our study has limitations. First, we used a claims-based algorithm to categorize mammograms as screening vs diagnostic, which may result in some misclassification. Efforts are under way to incorporate method of detection into cancer registry data, which could help address this limitation in future research.^25,26,27,28^ Second, we were unable to fully account for the effect that factors such as breast density and family history may have on disease extent at diagnosis, as these data are not available in the SEER-Medicare dataset.^13^ However, we controlled for demographic, biological, and social factors that may confound the association of screening history with stage and mortality.^29,30,31,32,33^ Additionally, the benefits of prior screening could be subject to lead-time bias, with the time from diagnosis to death being extended in this group due to earlier detection of nonsymptomatic disease.

## Conclusions

This cohort study found that prior screening mammography was associated with earlier stage at breast cancer diagnosis and lower breast cancer mortality in women aged 70 years and older with screen-detected breast cancers. Our findings support the potential for routine screening to improve breast cancer outcomes. However, our findings may have been limited by the residual confounding due to differences between individuals who underwent screening and those who did not.

## Notes

Supplement 1. **eMethods.** Algorithm to Identify Screen-Detected Breast Cancer **eTable 1.** CPT/HCPCS and *ICD-9/10* Codes Used to Identify Breast Cancer Screening and Primary Care Use **eTable 2.** Main Analysis: Adjusted Associations Between History of Screening and Other Covariates and Stage at Diagnosis; Patient Factors and History of Screening; and History of Screening and Breast Cancer Mortality **eTable 3.** Sensitivity Analysis I: Among Women Aged ≥75, Adjusted Associations Between History of Screening, Stage at Diagnosis, and Breast Cancer–Specific Mortality **eTable 4.** Sensitivity Analysis II: Among Women With Surgery, Adjusted Association Between History of Screening and Breast Cancer–Specific Mortality **eTable 5.** Additional Analysis I: Among Women With Prior Screening, Adjusted Associations Between the Number of Prior Screenings, the Time to Most Recent Screening, Stage at Diagnosis, and Breast Cancer–Specific Mortality **eTable 6.** Additional Analysis II: Among Women With Prior Screening, Adjusted Associations Between History of Abnormal Screening, Stage at Diagnosis, and Breast Cancer–Specific Mortality

Supplement 2.
Data Sharing Statement
