# Supplementary material for: Screening History, Stage at Diagnosis, and Mortality in Screen-Detected Breast Cancer
Source: JAMA Netw Open. 2025 Apr 15;8(4):e255322. doi: 10.1001/jamanetworkopen.2025.5322 (PMC12000969; doi:10.1001/jamanetworkopen.2025.5322)
Supplement: Supplement 1. — eMethods. Algorithm to Identify Screen-Detected Breast Cancer eTable 1. CPT/HCPCS and ICD-9/10 Codes Used to Identify Breast Cancer Screening and Primary Care Use eTable 2. Main Analysis: Adjusted Associations Between History of Screening and Other Covariates and Stage at Diagnosis; Patient Factors and History of Screening; and History of Screening and Breast Cancer Mortality eTable 3. Sensitivity Analysis I: Among Women Aged ≥75, Adjusted Associations Between History of Screening, Stage at Diagnosis, and Breast Cancer–Specific Mortality eTable 4. Sensitivity Analysis II: Among Women With Surgery, Adjusted Association Between History of Screening and Breast Cancer–Specific Mortality eTable 5. Additional Analysis I: Among Women With Prior Screening, Adjusted Associations Between the Number of Prior Screenings, the Time to Most Recent Screening, Stage at Diagnosis, and Breast Cancer–Specific Mortality eTable 6. Additional Analysis II: Among Women With Prior Screening, Adjusted Associations Between History of Abnormal Screening, Stage at Diagnosis, and Breast Cancer–Specific Mortality [file jamanetwopen-e255322-s001.pdf]

Supplemental Online Content

Huang S, Westvold SJ, Soulos PR, et al. Screening history, stage at diagnosis, and mortality in screen-detected breast cancer. *JAMA Netw Open*. 2025;8(4):e255322. doi:10.1001/jamanetworkopen.2025.5322

eMethods. Algorithm to Identify Screen-Detected Breast Cancer

eTable 1. CPT/HCPCS and *ICD-9/10* Codes Used to Identify Breast Cancer Screening and Primary Care Use

eTable 2. Main Analysis: Adjusted Associations Between History of Screening and Other Covariates and Stage at Diagnosis; Patient Factors and History of Screening; and History of Screening and Breast Cancer Mortality

eTable 3. Sensitivity Analysis I: Among Women Aged ⩾75, Adjusted Associations Between History of Screening, Stage at Diagnosis, and Breast Cancer–Specific Mortality

eTable 4. Sensitivity Analysis II: Among Women With Surgery, Adjusted Association Between History of Screening and Breast Cancer–Specific Mortality

eTable 5. Additional Analysis I: Among Women With Prior Screening, Adjusted Associations Between the Number of Prior Screenings, the Time to Most Recent Screening, Stage at Diagnosis, and Breast Cancer–Specific Mortality

eTable 6. Additional Analysis II: Among Women With Prior Screening, Adjusted Associations Between History of Abnormal Screening, Stage at Diagnosis, and Breast Cancer–Specific Mortality

This supplemental material has been provided by the authors to give readers additional information about their work.

© 2025 Huang S et al. *JAMA Network Open*.

eMethods.

1. Identifying screen-detected breast cancer

We used a validated claims-based algorithm developed by Fenton et al. (2014) to distinguish screen-detected from non-screen detected breast cancer.[1] The algorithm was developed to distinguish screening mammograms from diagnostic mammograms. Not all women included in this 2014 validation study had a known diagnosis of breast cancer. As our study population is women with screen-detected breast cancer and does not include a non-cancer sample, we looked for a screening mammogram (as identified by the algorithm) that occurred within 123 days prior to their breast cancer diagnosis. This 123-day window was established by a subsequent algorithm validated by Fenton et al. (2016) that distinguishes breast cancers detected at screening mammography.[2] We used this component of the 2016 algorithm as neither of the two algorithms start with a known breast cancer diagnosis to determine if it was screen or non-screening detected disease. If the mammogram that occurred within 123 days of breast cancer diagnosis was a screening mammogram per the Fenton (2014) algorithm, we considered this to be screen-detected breast cancer. This approach of combining elements from these two validated claims-based algorithms was used in our prior work.[3]

© 2025 Huang S et al. *JAMA Network Open*.

**eTable 1. Codes used to identify screening history and primary care use**	
**Codes used to distinguish screen-detected vs non-screen detected breast cancers**	
**Detection Mode**	**CPT/HCPCS Codes (Unless otherwise noted)**
Screening Mammography	'76092','G0202','G0203','77057','77067', '77052','76083','76085','77063'
Diagnostic Mammography	'76091','G0204','G0205','77056','77066', '76090','77055','77065','G0206','G0207', '76082','77051','77061','77062','G0279','G0236'
Breast Symptoms	‘6117’, 'N644','N6451','N6452','N6453','N6459'
**Codes used to distinguish abnormal vs non-abnormal screenings**	
Abnormal Mammography	ICD-9-CM and ICD-10-CM Codes: ‘7938’, ‘R920’, ‘R921’, ‘R922’, ‘R923’, ‘R928’

**Codes to identify primary care use**			
**Type of Primary Care**	**HCPCS**	**CPT**	**ICD-9/10**
Annual Wellness Visit[1]	G0402, G0468, G0438, G0439		
Flu Shot	G0008, Q2034, Q2035, Q2036, Q2037, Q2038, Q2039	90658, 90661,90660,90662, 90653,90654,90655, 90657,90656,90630,90664, 90666,90672,90673,90674,	9952, 3E01340, 3E02340

© 2025 Huang S et al. *JAMA Network Open*.

		90682,90685, 90687,90686, 90688,90689	
Primary Care Physician Visit	G0402, G0468, G0438, G0439	99202, 99203, 99204, 99205, 99212, 99213, 99214, 99215, 99387, 99397	

Footnotes:

[1] Annual wellness visit is a yearly preventive benefit of Medicare created in 2011.[4]

© 2025 Huang S et al. *JAMA Network Open*.

eTable 2. Adjusted associations between history of screening and other covariates and stage at diagnosis; patient factors and history of screening; and history of screening and breast cancer mortality. PCP visit = Primary Care Physician visit; AWV = Annual Wellness Visit; CI = Confidence Interval.

	**Later-Stage Diagnosis vs Very-Early-Stage Diagnosis (N=13,028)**		**BC Mortality vs Censored (N=13,028)**		**With Prior Scrning vs Without Prior Scrning (N=13,028)**	
	**Final Model**		**Final Model**		**Final Model**	
**Characteristic/Factor**	OR	95% CI	HR	95% CI	OR	95% CI
**Screening history**						
No prior screening	*Ref*		*Ref*		*NA*	
With prior screening	0.46	(0.42, 0.50)	0.63	(0.52, 0.76)		
**Age at diagnosis**						
70-74	*Ref*		*Ref*		*Ref*	
75-79	0.97	(0.88, 1.08)	1.44	(1.16, 1.79)	1.02	(0.92, 1.14)
80-84	1.10	(0.98, 1.23)	1.46	(1.15, 1.86)	0.92	(0.81, 1.04)
85+	1.12	(0.98, 1.29)	2.28	(1.76, 2.96)	0.50	(0.43, 0.57)
**Race & ethnicity**						
Non-Hispanic White	*Ref*		*Ref*		*Ref*	
Non-Hispanic Black	1.07	(0.90, 1.26)	0.93	(0.68, 1.27)	1.21	(1.01, 1.45)
Asian	1.1	(0.89, 1.37)	0.93	(0.58, 1.47)	0.91	(0.72, 1.15)
Hispanic	1.09	(0.89, 1.33)	0.75	(0.47, 1.18)	0.93	(0.76, 1.14)
Other/Unknown	1.01	(0.74, 1.38)	1.23	(0.67, 2.27)	1.09	(0.77, 1.54)
**Medicare/Medicaid dual eligible**						
No	*Ref*		*Ref*		*Ref*	
Yes	1.17	(1.02, 1.33)	1.02	(0.79, 1.31)	0.52	(0.45, 0.59)
**Area-level percent of residents with poverty**						
0-<5%	*Ref*		*Ref*		*Ref*	

© 2025 Huang S et al. *JAMA Network Open*.

5-10%	1.12	(1.00, 1.25)	1.21	(0.94, 1.55)	0.95	(0.84, 1.08)
10-<20%	1.12	(0.99, 1.27)	1.53	(1.18, 1.98)	1.01	(0.89, 1.15)
20-100%	1.21	(1.04, 1.41)	1.51	(1.11, 2.06)	0.99	(0.84, 1.17)
**Area-level percent of residents with less than high school degree**						
0-<30%	*Ref*		*Ref*		*Ref*	
30-<40%	0.83	(0.74, 0.93)	1.22	(0.96, 1.55)	0.90	(0.79, 1.02)
40-<50%	0.87	(0.77, 0.99)	1.05	(0.81, 1.35)	0.79	(0.70, 0.91)
50-100%	0.93	(0.82, 1.05)	1.04	(0.80, 1.34)	0.77	(0.68, 0.88)
**Marital status**						
Single/Unmarried	*Ref*		*Ref*		*Ref*	
Married	0.98	(0.90, 1.07)	0.95	(0.79, 1.15)	1.33	(1.21, 1.46)
Missing	0.81	(0.66, 0.99)	1.20	(0.83, 1.74)	0.94	(0.77, 1.15)
**Any PCP visit or AWV in the 5 years prior to diagnosis**						
No	*Ref*		*Ref*		*Ref*	
Yes	0.87	(0.71, 1.07)	1.03	(0.72, 1.46)	3.45	(2.85, 4.18)
**Flu shots in the 5 years prior to diagnosis**						
0	*NA*		*Ref*		*Ref*	
1-2	0.96	(0.84, 1.09)	1.00	(0.77, 1.30)	1.38	(1.21, 1.57)
3+	0.96	(0.87, 1.07)	0.96	(0.78, 1.19)	2.53	(2.28, 2.82)
**Grade**						
I	*Ref*		*Ref*		*NA*	
II	2.09	(1.91, 2.30)	1.42	(1.14, 1.77)		
III or IV	4.90	(4.30, 5.60)	2.23	(1.71, 2.90)		
**Histology**						
Ductal	*Ref*		*Ref*		*NA*	

© 2025 Huang S et al. *JAMA Network Open*.

Lobular	2.73	(2.46, 3.03)	1.20	(0.97, 1.49)		
Mixed Ductal and Lobular	1.72	(1.48, 2.00)	1.01	(0.72, 1.42)		
**Frailty**						
No	*Ref*		*Ref*		*Ref*	
Yes	1.05	(0.93, 1.17)	1.31	(1.05, 1.63)	0.64	(0.57, 0.73)
**Elixhauser comorbidity index**						
0	*Ref*		*Ref*		*Ref*	
1-2	1.00	(0.91, 1.10)	1.08	(0.88, 1.32)	1.08	(0.98, 1.20)
>=3	1.13	(1.00, 1.28)	1.24	(0.95, 1.61)	0.94	(0.82, 1.07)
**Received surgery**						
Received	*NA*		*Ref*		*NA*	
Not Received			3.31	(2.49, 4.39)		
**AJCC overall stages**						
I	*NA*		*Ref*		*NA*	
II			2.49	(2.02, 3.07)		
III			7.93	(6.16, 10.21)		
IV			23.32	(16.54, 32.89)		
**Year of diagnosis**						
2010	*Ref*		*Ref*		*Ref*	
2011	0.95	(0.81, 1.11)	1.05	(0.80, 1.38)	0.97	(0.81, 1.16)
2012	0.96	(0.82, 1.13)	1.19	(0.89, 1.58)	0.89	(0.75, 1.06)
2013	1.02	(0.87, 1.20)	1.00	(0.74, 1.35)	0.87	(0.73, 1.03)
2014	0.89	(0.76, 1.05)	0.88	(0.62, 1.23)	0.79	(0.66, 0.94)
2015	0.99	(0.85, 1.16)	0.82	(0.59, 1.16)	0.87	(0.73, 1.04)
2016	0.90	(0.77, 1.06)	0.70	(0.48, 1.02)	0.84	(0.70, 0.99)

© 2025 Huang S et al. *JAMA Network Open*.

2017	0.91	(0.78, 1.07)	0.76	(0.50, 1.16)	0.83	(0.69, 0.98)

© 2025 Huang S et al. *JAMA Network Open*.

eTable 3. Sensitivity analysis I: Among women aged 75+, adjusted associations between history of screening, stage at diagnosis, and breast cancer specific mortality. PCP visit = Primary Care Physician visit; AWV = Annual Wellness Visit; CI = Confidence Interval.

	**Later-Stage Diagnosis vs Early-Stage Diagnosis (N=8,013)**		**BC Mortality vs Censored (N=8,013)**	
	**Final Model**		**Final Model**	
**Characteristic/Factor**	OR	95% CI	HR	95% CI
**Screening History**				
No prior screening	*Ref*		*Ref*	
With prior screening	0.41	(0.36, 0.46)	0.56	(0.45, 0.69)
**Age**				
75-79	*Ref*		*Ref*	
80-84	1.12	(0.99, 1.26)	1.03	(0.82, 1.31)
85+	1.11	(0.97, 1.28)	1.59	(1.24, 2.06)
**Race & Ethnicity**				
Non-Hispanic White	*Ref*		*Ref*	
Non-Hispanic Black	1.13	(0.91, 1.41)	1.03	(0.71, 1.50)
Asian	1.06	(0.80, 1.40)	1.06	(0.64, 1.76)
Hispanic	1.01	(0.78, 1.30)	0.90	(0.53, 1.51)
Other/Unknown	0.73	(0.46, 1.17)	1.23	(0.57, 2.66)
**Area level % with less than high school degree**				
0-<30%	*Ref*		*Ref*	
30-<40%	0.77	(0.66, 0.89)	1.24	(0.93, 1.65)
40-<50%	0.89	(0.76, 1.04)	1.08	(0.80, 1.45)
50-100%	0.93	(0.79, 1.09)	1.12	(0.83, 1.51)
**Area level poverty**				
0-<5%	*Ref*		*Ref*	
5-10%	1.14	(0.99, 1.32)	1.08	(0.80, 1.46)
10-<20%	1.14	(0.97, 1.33)	1.43	(1.06, 1.93)
20-100%	1.20	(0.99, 1.45)	1.43	(1.00, 2.05)
**Medicare/Medicaid dual eligible**				
No	*Ref*		*Ref*	
Yes	1.17	(0.99, 1.38)	0.93	(0.69, 1.26)
**Marital status**				
Single/Unmarried	*Ref*		*Ref*	
Married	0.91	(0.82, 1.02)	1.01	(0.81, 1.26)
Missing	0.84	(0.66, 1.08)	1.14	(0.73, 1.77)
**Any PCP visit or AWV in the 5 years prior to diagnosis**				
No	*Ref*		*Ref*	
Yes	0.84	(0.64, 1.09)	1.18	(0.77, 1.81)
**Flu shots in the 5 years prior to diagnosis**				
0	*Ref*		*Ref*	
1-2	0.94	(0.79, 1.12)	1.03	(0.76, 1.41)
3+	1.02	(0.89, 1.17)	0.88	(0.69, 1.14)
**Grade**				
I	*Ref*		*Ref*	
II	2.12	(1.89, 2.39)	1.35	(1.05, 1.74)
III or IV	5.14	(4.33, 6.10)	2.08	(1.53, 2.83)
**Histology**				
Ductal	*Ref*		*Ref*	
Lobular	2.91	(2.54, 3.33)	1.04	(0.81, 1.35)
Mixed Ductal and Lobular	1.81	(1.49, 2.20)	0.98	(0.65, 1.47)
**Frail**				
No	*Ref*		*Ref*	
Yes	1.12	(0.97, 1.29)	1.41	(1.09, 1.81)
**Elixhauser comorbidity group**				
1	*Ref*		*Ref*	
2	0.94	(0.83, 1.06)	1.06	(0.83, 1.35)

© 2025 Huang S et al. *JAMA Network Open*.

3	1.02	(0.88, 1.19)	1.16	(0.85, 1.57)
**Received surgery**				
Received	*NA*		*Ref*	
Not Received			3.52	(2.58, 4.80)
**AJCC overall stages**				
I	*NA*		*Ref*	
II			2.20	(1.73, 2.81)
III			6.67	(4.92, 9.04)
IV			18.25	(12.21, 27.26)
**Year of diagnosis**				
2010	*Ref*		*Ref*	
2011	0.97	(0.79, 1.18)	0.95	(0.69, 1.33)
2012	0.95	(0.77, 1.16)	1.13	(0.81, 1.59)
2013	1.01	(0.82, 1.23)	0.97	(0.68, 1.37)
2014	0.94	(0.76, 1.16)	0.84	(0.57, 1.25)
2015	1.05	(0.85, 1.28)	0.78	(0.52, 1.16)
2016	0.93	(0.76, 1.13)	0.68	(0.44, 1.06)
2017	0.97	(0.79, 1.19)	0.71	(0.43, 1.17)

© 2025 Huang S et al. *JAMA Network Open*.

eTable 4. Sensitivity analysis II: Among women with surgery, adjusted association between history of screening and breast cancer specific mortality. PCP visit = Primary Care Physician visit; AWV = Annual Wellness Visit; CI = Confidence Interval.

	BC Mortality vs Censored (N=12,628)	
	Final Model	
**Characteristic/Factor**	HR	95% CI
**Screening History**		
No Prior Screening	*Ref*	
With Prior Screening	0.65	(0.53, 0.80)
**Age at diagnosis**		
70-74	*Ref*	
75-79	1.48	(1.18, 1.87)
80-84	1.34	(1.02, 1.76)
85+	2.50	(1.88, 3.32)
**Race & ethnicity**		
Non-Hispanic White	*Ref*	
Non-Hispanic Black	1.03	(0.73, 1.45)
Asian	1.06	(0.64, 1.73)
Hispanic	0.67	(0.40, 1.12)
Other/Unknown	1.10	(0.52, 2.33)
**Area level % with less than high school degree**		
0-<30%	*Ref*	
30-<40%	1.25	(0.95, 1.63)
40-<50%	1.07	(0.80, 1.43)
50-100%	1.12	(0.85, 1.49)
**Area level poverty**		
0-<5%	*Ref*	
5-10%	1.18	(0.89, 1.56)
10-<20%	1.49	(1.12, 1.98)
20-100%	1.42	(1.01, 2.01)
**Medicare/Medicaid dual eligible**		
No	*Ref*	
Yes	0.98	(0.73, 1.31)
**Marital status**		
Single/Unmarried	*Ref*	
Married	1.02	(0.84, 1.25)
Missing	1.27	(0.85, 1.91)
**Histology**		
Ductal	*Ref*	
Lobular	1.36	(1.08, 1.72)
Mixed Ductal and Lobular	0.89	(0.61, 1.29)
**Elixhauser comorbidity group**		
1	*Ref*	
2	1.11	(0.89, 1.38)
3	1.31	(0.99, 1.73)
**Frailty**		
No	*Ref*	
Yes	1.26	(0.98, 1.61)
**Any PCP visit or AWV in the past 5 years**		

© 2025 Huang S et al. *JAMA Network Open*.

No	*Ref*	
Yes	0.83	(0.55, 1.23)
**Flu shots in the past 5 Years**		
0	*Ref*	
1-2	1.10	(0.82, 1.47)
3+	0.97	(0.76, 1.23)
**AJCC Overall Stages**		
I	*Ref*	
II	2.57	(2.07, 3.21)
III	8.02	(6.14, 10.48)
IV	34.98	(22.53, 54.32)
**Grade**		
I	*Ref*	
II	1.46	(1.15, 1.86)
III or IV	2.40	(1.80, 3.20)
**Year of diagnosis**		
2010	*Ref*	
2011	0.98	(0.73, 1.32)
2012	0.99	(0.73, 1.35)
2013	0.88	(0.63, 1.21)
2014	0.76	(0.52, 1.09)
2015	0.78	(0.53, 1.13)
2016	0.70	(0.46, 1.06)
2017	0.67	(0.40, 1.10)

© 2025 Huang S et al. *JAMA Network Open*.

eTable 5. Additional analysis I: Among women with prior screening, adjusted associations between the number of prior screenings, the time to most recent screening, stage at diagnosis, and breast cancer specific mortality. PCP visit = Primary Care Physician visit; AWV = Annual Wellness Visit; CI = Confidence Interval.

	**Later-Stage Diagnosis vs Early-Stage Diagnosis (N=10,094)**		**BC Mortality vs Censored (N=10,094)**	
	**Final Model**		**Final Model**	
**Characteristic/Factor**	OR	95% CI	HR	95% CI
**Number of prior screenings**				
One prior screening	*Ref*		*Ref*	
Two prior screenings	0.73	(0.63, 0.84)	0.90	(0.66, 1.23)
Three or four prior screenings	0.68	(0.58, 0.79)	0.63	(0.44, 0.89)
**Time to the most recent screening from diagnosis**				
More than 2 years	*Ref*		*Ref*	
Less than or equal to 2 years	1.06	(0.92, 1.22)	1.04	(0.77, 1.42)
**Age at diagnosis**				
70-74	*Ref*		*Ref*	
75-79	0.94	(0.84, 1.05)	1.28	(0.98, 1.68)
80-84	1.00	(0.88, 1.15)	1.24	(0.90, 1.70)
85+	0.95	(0.80, 1.12)	2.29	(1.62, 3.22)
**Race & Ethnicity**				
Non-Hispanic White	*Ref*		*Ref*	
Non-Hispanic Black	1.04	(0.85, 1.28)	1.14	(0.75, 1.75)
Asian	1.24	(0.97, 1.60)	0.74	(0.39, 1.40)
Hispanic	1.27	(1.00, 1.62)	0.54	(0.26, 1.10)
Other/Unknown	0.98	(0.68, 1.40)	1.36	(0.60, 3.07)
**Area level % with less than high school degree**				
0-<30%	*Ref*		*Ref*	
30-<40%	0.79	(0.69, 0.90)	1.16	(0.86, 1.57)
40-<50%	0.85	(0.73, 0.98)	0.79	(0.55, 1.12)
50-100%	0.93	(0.80, 1.07)	0.98	(0.71, 1.36)
**Area level poverty**				
0-<5%	*Ref*		*Ref*	
5-10%	1.22	(1.07, 1.39)	1.01	(0.73, 1.40)
10-<20%	1.15	(0.99, 1.32)	1.34	(0.97, 1.85)
20-100%	1.22	(1.02, 1.46)	1.23	(0.82, 1.84)
**Medicare/Medicaid dual eligible**				
No	*Ref*		*Ref*	
Yes	1.00	(0.84, 1.19)	1.07	(0.74, 1.55)
**Marital status**				
Single/Unmarried	*Ref*		*Ref*	
Married	1.01	(0.91, 1.12)	0.89	(0.70, 1.12)
Missing	0.79	(0.62, 1.01)	0.96	(0.56, 1.63)
**Any PCP visit or AWV in the 5 years prior to diagnosis**				
No	*Ref*		*Ref*	
Yes	1.08	(0.79, 1.49)	0.87	(0.44, 1.70)
**Flu shots in the 5 years prior to diagnosis**				
0	*Ref*		*Ref*	
1-2	0.98	(0.83, 1.16)	1.10	(0.76, 1.60)
3+	1.00	(0.88, 1.13)	1.09	(0.81, 1.47)
**Grade**				
I	*Ref*		*Ref*	
II	2.05	(1.84, 2.28)	1.54	(1.16, 2.04)
III or IV	4.48	(3.83, 5.23)	2.66	(1.89, 3.75)
**Histology**				
Ductal	*Ref*		*Ref*	
Lobular	2.82	(2.50, 3.18)	1.42	(1.09, 1.86)
Mixed Ductal and Lobular	1.83	(1.54, 2.17)	0.57	(0.32, 1.00)
**Frailty**				
No	*Ref*		*Ref*	
Yes	1.00	(0.87, 1.15)	1.36	(1.00, 1.84)
**Elixhauser comorbidity group**				
0	*Ref*		*Ref*	
1-2	0.97	(0.87, 1.08)	1.11	(0.85, 1.43)
3+	1.16	(1.00, 1.34)	1.23	(0.87, 1.74)
**Received surgery**				

© 2025 Huang S et al. *JAMA Network Open*.

Received	*NA*		*Ref*	
Not Received			2.87	(1.85, 4.47)
**AJCC overall stages**				
I	*NA*		*Ref*	
II			2.18	(1.67, 2.84)
III			7.92	(5.72, 10.97)
IV			26.69	(16.59, 42.93)
**Year of diagnosis**				
2011	0.96	(0.79, 1.15)	1.12	(0.79, 1.60)
2012	0.98	(0.81, 1.18)	1.17	(0.80, 1.69)
2013	0.98	(0.81, 1.18)	1.02	(0.68, 1.53)
2014	0.92	(0.76, 1.12)	1.00	(0.65, 1.53)
2015	0.99	(0.82, 1.19)	0.85	(0.55, 1.33)
2016	0.92	(0.76, 1.10)	0.71	(0.42, 1.20)
2017	0.88	(0.73, 1.06)	0.94	(0.54, 1.63)

© 2025 Huang S et al. *JAMA Network Open*.

eTable 6. Additional analysis II: Among women with prior screening, adjusted associations between history of abnormal screening, stage at diagnosis, and breast cancer specific mortality. PCP visit = Primary Care Physician visit; AWV = Annual Wellness Visit; CI = Confidence Interval.

	Later-Stage Diagnosis vs Early-Stage Diagnosis (N=10,094)		BC Mortality vs Censored (N=10,094)	
	Final Model		Final Model	
**Characteristic/Factor**	OR	95% CI	HR	95% CI
**Screening History**				
Normal prior screening	*Ref*		*Ref*	
Any of the prior screenings was abnormal	1.03	(0.92, 1.14)	1.00	(0.79, 1.29)
**Age at diagnosis**				
70-74	*Ref*		*Ref*	
75-79	0.94	(0.84, 1.05)	1.26	(0.96, 1.65)
80-84	0.99	(0.87, 1.13)	1.21	(0.88, 1.66)
85+	0.94	(0.80, 1.12)	2.27	(1.61, 3.20)
**Race & Ethnicity**				
Non-Hispanic White	*Ref*		*Ref*	
Non-Hispanic Black	1.03	(0.84, 1.26)	1.14	(0.74, 1.74)
Asian	1.25	(0.98, 1.61)	0.74	(0.39, 1.39)
Hispanic	1.28	(1.00, 1.63)	0.54	(0.26, 1.11)
Other/Unknown	0.98	(0.68, 1.42)	1.34	(0.59, 3.02)
**Area level % with less than high school degree**				
0-<30%	*Ref*		*Ref*	
30-<40%	0.79	(0.69, 0.90)	1.17	(0.86, 1.58)
40-<50%	0.85	(0.74, 0.98)	0.80	(0.56, 1.13)
50-100%	0.93	(0.81, 1.08)	0.97	(0.70, 1.34)
**Area level poverty**				
0-<5%	*Ref*		*Ref*	
5-10%	1.23	(1.08, 1.40)	1.05	(0.76, 1.44)
10-<20%	1.16	(1.01, 1.34)	1.37	(0.99, 1.89)
20-100%	1.23	(1.03, 1.48)	1.30	(0.87, 1.94)
**Medicare/Medicaid dual eligible**				
No	*Ref*		*Ref*	
Yes	1.04	(0.88, 1.24)	1.13	(0.78, 1.63)
**Marital status**				
Single/Unmarried	*Ref*		*Ref*	
Married	1.01	(0.91, 1.11)	0.86	(0.68, 1.09)
Missing	0.80	(0.63, 1.02)	0.95	(0.56, 1.62)
**Any PCP visit or AWV in the 5 years prior to diagnosis**				
No	*Ref*		*Ref*	
Yes	1.08	(0.79, 1.49)	0.87	(0.44, 1.70)
**Flu shots in the 5 years prior to diagnosis**				
0	*Ref*		*Ref*	
1-2	0.98	(0.83, 1.16)	1.06	(0.73, 1.54)
3+	0.96	(0.85, 1.09)	1.03	(0.77, 1.38)
**Grade**				
I	*Ref*		*Ref*	
II	2.04	(1.89, 2.39)	1.53	(1.16, 2.02)
III or IV	4.51	(4.33, 6.10)	2.66	(1.89, 3.75)
**Histology**				
Ductal	*Ref*		*Ref*	
Lobular	2.80	(2.48, 3.15)	1.39	(1.06, 1.82)
Mixed Ductal and Lobular	1.82	(1.53, 2.17)	0.56	(0.32, 0.98)
**Frailty**				
No	*Ref*		*Ref*	
Yes	1.01	(0.88, 1.17)	1.38	(1.02, 1.88)
**Elixhauser comorbidity group**				
0	*Ref*		*Ref*	
1-2	0.97	(0.88, 1.09)	1.13	(0.87, 1.46)
3+	1.17	(1.01, 1.35)	1.25	(0.89, 1.77)
**Received surgery**				
Received	*NA*		*Ref*	
Not Received			2.83	(1.81, 4.42)
**AJCC overall stages**				
I	*NA*		*Ref*	
II			2.23	(1.71, 2.91)
III			8.23	(5.95, 11.40)
IV			26.55	(16.43, 42.91)

© 2025 Huang S et al. *JAMA Network Open*.

**Year of diagnosis**				
2010	*Ref*		*Ref*	
2011	0.96	(0.80, 1.15)	1.15	(0.81, 1.64)
2012	0.98	(0.81, 1.18)	1.18	(0.81, 1.71)
2013	0.98	(0.81, 1.18)	1.03	(0.69, 1.54)
2014	0.94	(0.77, 1.13)	1.05	(0.69, 1.61)
2015	0.99	(0.83, 1.19)	0.87	(0.56, 1.37)
2016	0.92	(0.77, 1.11)	0.71	(0.42, 1.20)
2017	0.88	(0.73, 1.07)	0.92	(0.53, 1.61)

© 2025 Huang S et al. *JAMA Network Open*.

1. Fenton JJ, Zhu W, Balch S, Smith-Bindman R, Fishman P, Hubbard RA. Distinguishing screening from diagnostic mammograms using Medicare claims data. *Med Care*. Jul 2014;52(7):e44-51. doi:10.1097/MLR.0b013e318269e0f5
2. Fenton JJ, Onega T, Zhu W, et al. Validation of a Medicare Claims-based Algorithm for Identifying Breast Cancers Detected at Screening Mammography. *Med Care*. Mar 2016;54(3):e15-22. doi:10.1097/MLR.0b013e3182a303d7
3. Robinson TJ, Wilson LE, Marcom PK, et al. Analysis of Sociodemographic, Clinical, and Genomic Factors Associated With Breast Cancer Mortality in the Linked Surveillance, Epidemiology, and End Results and Medicare Database. *JAMA Netw Open*. Oct 1 2021;4(10):e2131020. doi:10.1001/jamanetworkopen.2021.31020
4. Colburn JL, Nothelle S. The Medicare Annual Wellness Visit. *Clin Geriatr Med*. Feb 2018;34(1):1-10. doi:10.1016/j.cger.2017.09.001

© 2025 Huang S et al. *JAMA Network Open*.
