# Supplementary material for: Screening History, Stage at Diagnosis, and Mortality in Screen-Detected Breast Cancer
Source: JAMA Netw Open. 2025 Apr 15;8(4):e255322. doi: 10.1001/jamanetworkopen.2025.5322 (PMC12000969; doi:10.1001/jamanetworkopen.2025.5322)
Supplement: Supplement 2. — Data Sharing Statement [file jamanetwopen-e255322-s002.pdf]

Data Sharing Statement

Huang. Mammographic Screening History, Stage at Diagnosis, and Mortality in Screen-Detected Breast Cancer. *JAMA Netw Open*. Published April 15, 2025.
doi:10.1001/jamanetworkopen.2025.5322

Data
Data available: Yes
Data types: Deidentified participant data, Data dictionary
How to access data: stark.huang@yale.edu
When available: With publication

Supporting Documents
Document types: None

Additional Information
Who can access the data: researchers whose proposed use of the data has been approved
Types of analyses: for a specified purpose
Mechanisms of data availability: with a signed data access agreement
